# Notes and News

**Published:** 1905-06-10

**Authors:** 


### Notes and News.

The Duchess of Albany will attend a Garden Fête in aid of the Royal Waterloo Hospital for Children and Women at the Manor House, Stoke D'Abernon, on Saturday, June 17th.

At the Royal Sanitary Institute on Friday, June 16th, at 4.30, Mr. Edwin T. Hall, F.R.I.B.A., will open a discussion on consumption sanatoria, from the point of design and locality.

The managers of the Falkirk Infirmary propose to extend the institution by the erection of a new administrative block, a new ward for six beds, new operating-room, and new lift. The estimated cost is £2,300.

The Middlesbrough Isolation Hospital, which was built in 1872, has recently been extended. Two pavilions have been added for 60 patients and a new hospital for small-pox has been connected with the system. The small-pox hospital will provide for 24 patients.

The new Montagu Hospital at Mexbro' has been opened. Mr. F. Montagu gave the site, valued at £750, and £1,500 in money, besides being most energetic in furthering the scheme. The sum expended amounted to £10,000, which has been mainly contributed by the collieries and private residents in the neighbourhood, £2,000 still being required. The accommodation is for about 26 adults in the general wards, and there is also a children's ward and isolation ward.

The Robert Arthington Hospital of Recovery has been opened at Cookridge, near Leeds. It fulfils a very useful purpose, for it tends both to economy and to the complete recovery of the patient. The new hospital will provide for 50 patients, and is an extension of the previously-named Ida Hospital. It has been paid for through a bequest of the late Mr. Robert Arthington, £12,000 of which was given to the Leeds Infirmary and expended on the new institution.

An appeal is being made for an increase of assistance from this country for the Japanese wounded.

An extraordinary epidemic of fainting amongst school-girls attending the Elementary Schools in Derbyshire, has occurred recently. Every effort to definitely trace the cause has failed.

The Birmingham, Aston, and King's Norton poor-law authorities are about to establish an institution for the care of the feeble-minded and epileptics. It occupies 128 acres, and the building is estimated at £13,500.

A very complete electro-therapeutic department is nearly equipped at the Norfolk and Norwich Hospital. This has been provided through the generosity of Mr. Eustace Gurney, who presented £1,000 to the Institution on his marriage. A good many improvements have been recently effected at the hospital.

The *British Medical Journal* for June 3rd contains information which will be of interest and importance to many young men entering the medical profession. It must be obvious to all when commencing a medical career, or very soon after, that the British Isles cannot give employment to anything like the whole number who are educated in the medical schools. Except for the most brilliant students, the prospect is often one of comparative poverty and monotony. There are many who dread the latter more than the former, and only want of knowledge prevents them seeking the variety of work under less familiar and limited conditions. The review we refer to is entitled "The Medical Profession Abroad," and it gives an opportunity for study and selection to the student who is inclined to practise away from the mother country.

Every year the railway companies offer fresh facilities to the holiday-maker, and they issue a mass of alluring literature affording information and stimulating interest. We have before us, firstly, the London and South-Western Railway's Guide to hotels, boarding-houses, and farmhouse apartments located within the range of their system. This Guide consists of 263 pages of matter, it is well illustrated, and contains a map of England indicating the various railways and the routes to the Continent. The information is of a most varied and comprehensive nature. The Great Eastern Railway send an attractive booklet entitled "Week End Holidays from London." This, too, is delightfully illustrated, and includes the official time tables in connection with the localities mentioned. From the same railway also comes a useful, well illustrated, and interesting tourist Guide to the Continent, as reached most conveniently by the routes in connection with this railway. This includes the Tyrol and Northern Europe. The District Council of Weston-super-Mare urge the claims of their town in a beautifully got up little work, which can be obtained free from the Town Clerk. The book contains a reference to the train service from all parts.

